# Etch and Print: Graphene-Based Diodes for Silicon Technology

**DOI:** 10.1021/acsnano.2c10684

**Published:** 2022-12-07

**Authors:** Alessandro Grillo, Zixing Peng, Aniello Pelella, Antonio Di Bartolomeo, Cinzia Casiraghi

**Affiliations:** † Department of Chemistry, University of Manchester, ManchesterM13 9PL, United Kingdom; ‡ Physics Department “E. R. Caianiello”, University of Salerno, via Giovanni Paolo II n. 132, Fisciano84084, Salerno, Italy

**Keywords:** inkjet printing, Schottky diodes, graphene−silicon junctions, photodiodes, back-end-of-line process

## Abstract

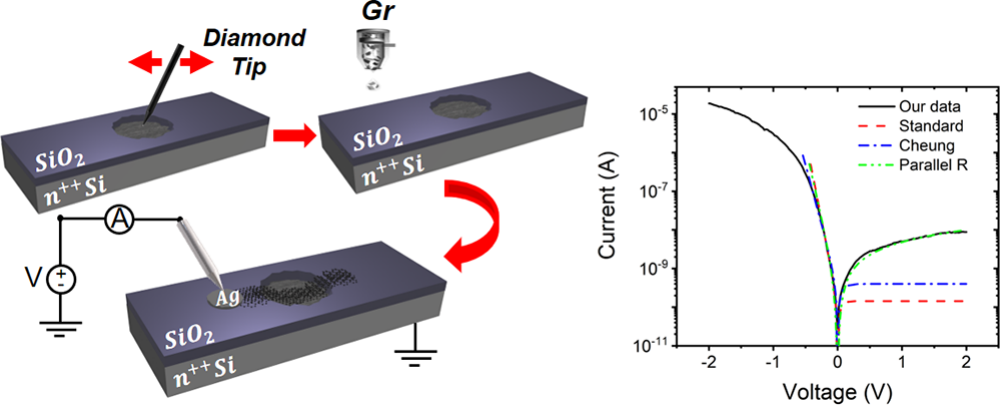

The graphene–silicon junction is one of the simplest conceivable interfaces in graphene-integrated semiconductor technology that can lead to the development of future generation of electronic and optoelectronic devices. However, graphene’s integration is currently expensive and time-consuming and shows several challenges in terms of large-scale device fabrication, effectively preventing the possibility of implementing this technology into industrial processes. Here, we show a simple and cost-effective fabrication technique, based on inkjet printing, for the realization of printed graphene–silicon rectifying devices. The printed graphene–silicon diodes show an ON/OFF ratio higher than 3 orders of magnitude and a significant photovoltaic effect, resulting in a fill factor of ∼40% and a photocurrent efficiency of ∼2%, making the devices suitable for both electronic and optoelectronic applications. Finally, we demonstrate large-area pixeled photodetectors and compatibility with back-end-of-line fabrication processes.

Silicon has dominated the semiconductor technology scene throughout the past century, and nowadays it is still the most widely used material in many electronic applications. However, the future generation of electronic devices does require the use of thinner materials with higher electronic performance, which cannot be achieved with traditional semiconducting bulk materials.^1^

The family of two-dimensional (2D) materials, in particular, graphene, represents a very attractive platform for the future generation of electronics and optoelectronics.^2−4^ In addition to the unique properties of these materials, their dimensionality and low-temperature integration enable easy integration in a standard Si CMOS process flow.^4^ Indeed, back-end-of-line (BEOL) process techniques, where graphene is used as last layer of the process, have been used to demonstrate graphene–Si prototypes, such as graphene gas sensors,^5^ optical receivers,^6^ and image sensor arrays.^7^

The graphene–silicon junction is one of the simplest conceivable devices in a graphene-integrated semiconductor technology. Not only does it offers a great opportunity to study the physics occurring at the interface between a 2D and a 3D material, but it also provides the possibility to realize electronic devices with outstanding properties.^8^ For this reason, in the past few years, several studies have reported the fabrication of graphene–silicon-based rectifying or barrier-variable devices,^9,10^ photovoltaic cells,^11^ bias-tunable photodetectors,^12,13^ chemical sensors,^14^ and Schottky-barrier-based field-effect transistors.^15^ Despite these promising results, there are still several challenges associated with optimizing the material integration and device performance.^3,4^ In particular, previous works did focus on graphene produced by chemical vapor deposition (CVD), whose integration into a silicon chip requires to finely control the material growth (grain-free graphene will provide better performance) and to minimize wrinkle formation and residue associated with the transfer process,^16,17^ hence enabling device reproducibility. These challenges not only make the integration process expensive in terms of time and money, but it also considerably limits the possibility of extending the fabrication process on a large scale, effectively preventing the possibility of implementing this technology in an industrial process.

In this framework, inkjet printing represents a very attractive technique for device fabrication because of its scalability and cost effectiveness.^18^ It also enables reduction of materials and energy consumption, limiting the number of processing steps and the waste production during the fabrication.^18^ Graphene and 2D materials can be easily formulated in inkjet printable inks, either by using liquid phase exfoliation (LPE)^19,20^ or electrochemical exfoliation.^21^ Many works have reported hybrid or fully printed electronic devices made of 2D materials,^20,22−26^ but to date, a printed graphene–silicon diode has never been reported in the literature.

In this work, we present a simple and cost-effective fabrication technique for the realization of printed graphene–silicon rectifying devices. Mechanical scratching is used to remove a small region of thermally grown SiO_2_ from the silicon substrate. Then graphene is inkjet-printed on the exposed area to allow the formation of the graphene–silicon junction. The obtained devices show a typical diode behavior with an ON/OFF ratio (measured in air and at room temperature) of about 3 orders of magnitude. Several theoretical models have been applied to study the transport mechanisms of the junction and to estimate the figures of merit of the diode, such as the Schottky barrier height and the ideality factor. Furthermore, the behavior of the devices at different temperatures and under light irradiation is investigated. A significant photovoltaic effect is observed, making the diode suitable for advanced optoelectronic applications, such as photodetectors able to work in the long-wavelength range. In this framework, we demonstrate a device made of a network of printed diodes made on the same substrate, which acts as a spatially selective photodetector; i.e., the device allows one to identify which area of the chip has been irradiated with white light through the measurement of the variation of current measured by different diodes. Finally, to demonstrate the ease of large-scale production through this technique and compatibility with Si technology in a BEOL process, we fabricated the same diode on a prepatterned silicon substrate, where the oxide layers were selectively removed with standard clean-room-based etching techniques.

In conclusion, we demonstrate that inkjet printing offers a simple way to integrate graphene into silicon-based electronics, which can be used to fabricate diodes with a high ON/OFF ratio and a strong photovoltaic response that make them suitable for optoelectronic applications.

## Results and Discussion

### Diode Fabrication and Characterization

**Figure 1:**
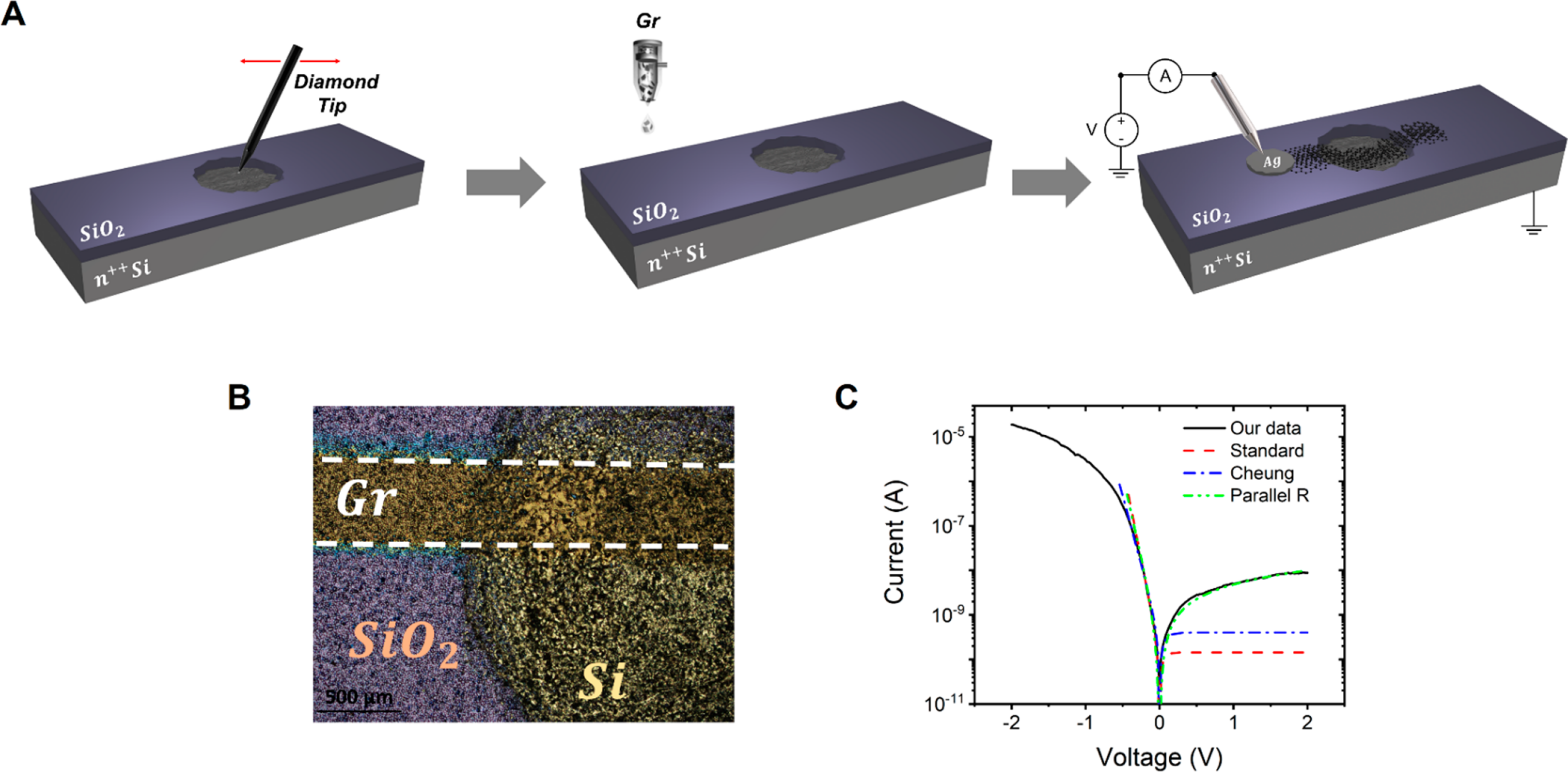
Schematic of the fabrication process and electrical characterization of the graphene-based diode. (A) Fabrication starts by scratching the substrate surface with a diamond tip to partially remove the SiO_2_ layer, then a graphene ink is printed onto the scratched area. Finally, the printed line is contacted with silver paste. (B) Optical image of the device showing a graphene line partially printed on the SiO_2_ substrate and partially overlapping the exposed Si area. Length and width of the graphene line are 5 and 0.5 mm, respectively. (C) *I*–*V* characteristic of the diode recorded in air and at room temperature (black line). Red, blue, and green dashed lines represent the fits obtained using the standard diode equation, the Cheung’s method parameters, and the modified model with the parallel resistance, respectively.

Devices were prepared on doped n-Si (100) wafers covered with 300 nm thermally grown SiO_2_ (see Methods). An area of about 7 mm^2^ was mechanically scratched trough the use of a diamond tip to locally remove the SiO_2_ and expose the Si surface (Figure 1A). A water-based graphene ink with concentration of 2 mg/mL was prepared as reported in ref (20) and was inkjet-printed on the exposed Si surface a few minutes after the scratch process, with 80 printing passes. After the printing, the devices were thermally annealed in vacuum at 300 °C for 1 h. The length and width of the graphene line are 5 and 0.5 mm, respectively, while the overlap area between graphene and silicon, i.e, the junction area, is 1.5 mm^2^, as shown in Figure 1B. We highlight that inkjet printing allows the junction area to be easily modified, where the smallest area achievable is only limited by the spatial resolution of the printer. The device was then contacted as shown in Figure 1A. Characterization of the printed graphene film morphology is shown in section I of the Supporting Information. Figure 1C shows the *I*–*V* characteristics of the Gr–Si device recorded under dark conditions at 300 K and atmosphere pressure. A typical diode behavior is observed with a rectification ratio (ON/OFF) of ∼10^3^ at *V* = ±2 V. The polarity of the diode is consistent with the formation of a n–n heterojunction between the slightly doped graphene^27^ and n-type silicon.^28^ Measurements have been performed on 12 samples, showing reproducible results. The *I*–*V* characteristics of all of the devices tested are reported in section II in the Supporting Information. Note that no rectifying behavior was observed when graphene was replaced by metallic electrodes (section II of the Supporting Information).

As first attempt, the experimental data are fitted with the standard diode equation:^28^
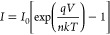
1where *I*_0_ is the reverse saturation current, *q* is the electron charge, *n* is the ideality factor, *k* is the Boltzmann constant, and *T* is the temperature. In the case of pure thermionic transport, *n* should be 1, but interfacial defects as well as unwanted insulating layers can lead to Schottky barrier inhomogeneities increasing the ideality factor.^29,30^ The fit of eq 1 (dashed red line in Figure 1C) shows a good agreement with the experimental data in the forward region up to *V* = −0.2 V. This is expected since in this range any series resistance can be neglected. The best fit is obtained with *I*_0_ = −1.4 × 10^–10^ A and *n* = 2.1. According to the thermionic emission theory, the reverse saturation current can be written as:^28^
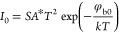
2where *S* is the area of the junction, A* is the Richardson constant (112 cm^–2^ K^–2^ for n-Si^31^), and φ_b__0_ is the Schottky barrier at zero voltage. By using *I*_0_ = −1.4 × 10^–10^ A, we obtain φ_b__0_ = 0.89 eV. This value seems too high and is likely overestimated due to the arbitrary choice of the fitting region and the A* value that can be quite different from the theoretical one in real devices. To improve the fit, we consider a more realistic model that takes into account the possible presence of a series resistance *R*_s_, which describes the diode current with the following equation:^32^
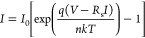
3Equation 3 can be used in conjunction with Cheung’s method^33^ to obtain the diode parameters: for *V* – *R*_s_*I* ≫ *nkT*/*q*, eq 3 can be written as:
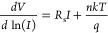
4and therefore can be used to evaluate *R*_s_ and *n*. Then defining the following function:^33^
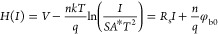
5it is possible to estimate the Schottky barrier height. From the linear fit of eqs 4 and 5, section II in the Supporting Information, we obtain *R*_s_ = 35 kΩ, *n* = 2.7, and φ_b__0_ = 0.28. The blue dashed curve in Figure 1C shows the experimental fit of eq 3, using the obtained fitting parameters, superimposed with the experimental data. A good agreement is obtained until the flat band condition is reached, i.e., *V* – *R*_s_*I* ≃ 0, where the diode equation (eq 3) does not apply anymore.

It is evident that both models fail in fitting the reverse current. To fix this, we consider a further improvement of eq 3 obtained adding a parallel resistance *R*_p_, which takes into account possible leakage currents through defects that might locally lower the barrier, the oxide, and the substrate edges, and given by:^30^
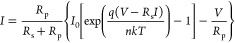
6The best fit is obtained with *R*_p_ = 195 MΩ and provides good agreement with both forward and reverse regions, as shown by the green dashed curve in Figure 1C.

**Figure 2:**
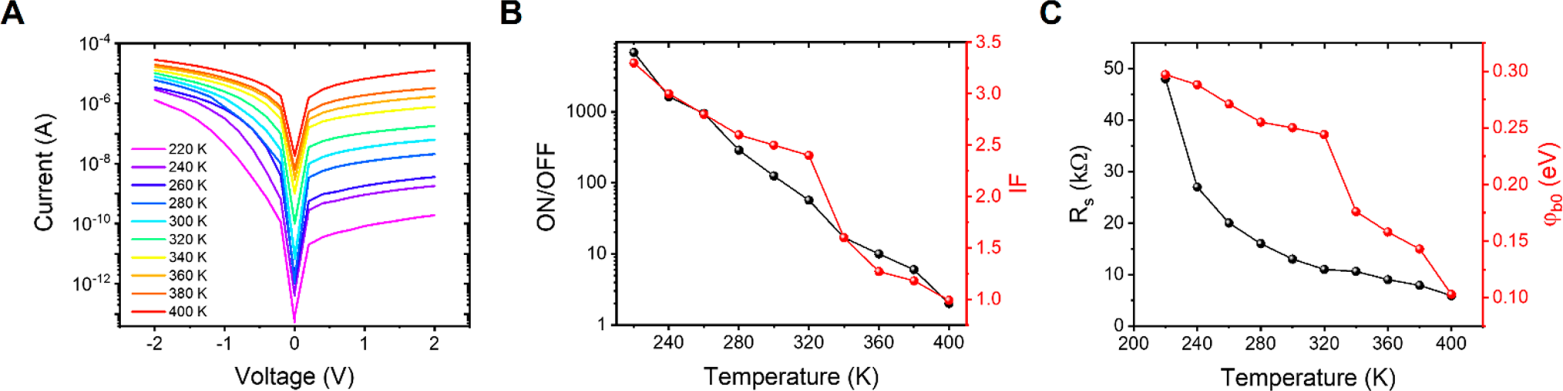
Electrical characterization of the diode at different temperatures. (A) *I*–*V* characteristics of the diode recorded in the 220–400 K range and at a pressure of 1 mbar. (B) ON/OFF ratio (black dots) and ideality factor (red dots) as a function of the temperature. (C) Series resistance (black dots) and Schottky barrier height (red dots) evaluated from the measurements as a function of the temperature.

To further investigate the conduction mechanisms of the diode, we study how the current in the devices is affected by the temperature. Figure 2A reports the *I*–*V* characteristics of the diodes recorded in the 220–400 K range. As predicted by the thermionic theory,^28^ an increase in both forward and reverse current is observed with an increase of the temperature. The reverse current is more affected by the temperature, causing a decrease in the ON/OFF ratio with increasing temperature, as shown in Figure 2B. Cheung’s method described above, applied to the forward characteristic, is used to estimate the values of *n*, *R*_s_, and φ_b__0_ at each temperature. The red dots in Figure 2B reveal that the ideality factor decreases with the temperature. This is due to the fact that at low temperatures the purely thermionic transport theory is no longer valid: other effects, such as tunneling and diffusion, can start to play a relevant role. Figure 2C (black dots) shows the series resistance variation as a function of temperature, revealing a typical semiconductive behavior, caused by both the silicon substrate and the printed graphene film. Then we observe that the height of the Schottky barrier decreases with increasing temperature, as also confirmed by the decrease in the ON/OFF ratio, as shown in Figure 2C (red dots). The obtained φ_b__0_ values are in the 0.1–0.3 eV range, which disagrees with the theoretical value based on the Schottky–Mott model according to which φ_b__0_ = ϕ_g_ – χ_Si_ = 0.5 eV, where ϕ_g_ = 4.54 eV is the commonly used work function of graphene^34,35^ and χ_Si_ = 4.05 eV is the electronic affinity of silicon^28^ (see band diagram of an undoped and pristine graphene/n-type silicon junction in section II in the Supporting Information). This discrepancy is likely due both to graphene being doped (n-doping would raise the Fermi level reducing its work function) and to a probable Fermi level pinning caused by the presence of defects or impurities at the junction interface, in agreement with what was observed in other works based on CVD graphene.^36−38^

The *I*–*V* characteristics at different temperatures have been used to construct the Richardson’s plot and to estimate the values of A* and φ_b__0_ through a different method (section II, Supporting Information). Although the origin of a lower effective Richardson constant has been attributed to different phenomena, such as the massless Dirac fermion nature of carriers in graphene,^39^ a Landauer transport mechanism^40^ or the presence of a native oxide layer at the Gr–Si interface,^30^ it can be seen the A* and φ_b__0_ obtained through Richardson’s method are very unrealistic as they are averaged on *I*–*V* curves in which the thermionic emission is not the only conduction mechanism, such as those at low temperatures. Hence, we can state that Cheung’s method gives a better estimate of the diode parameters among the models used.

### Photovoltaic Effect and Diodes Array

**Figure 3:**
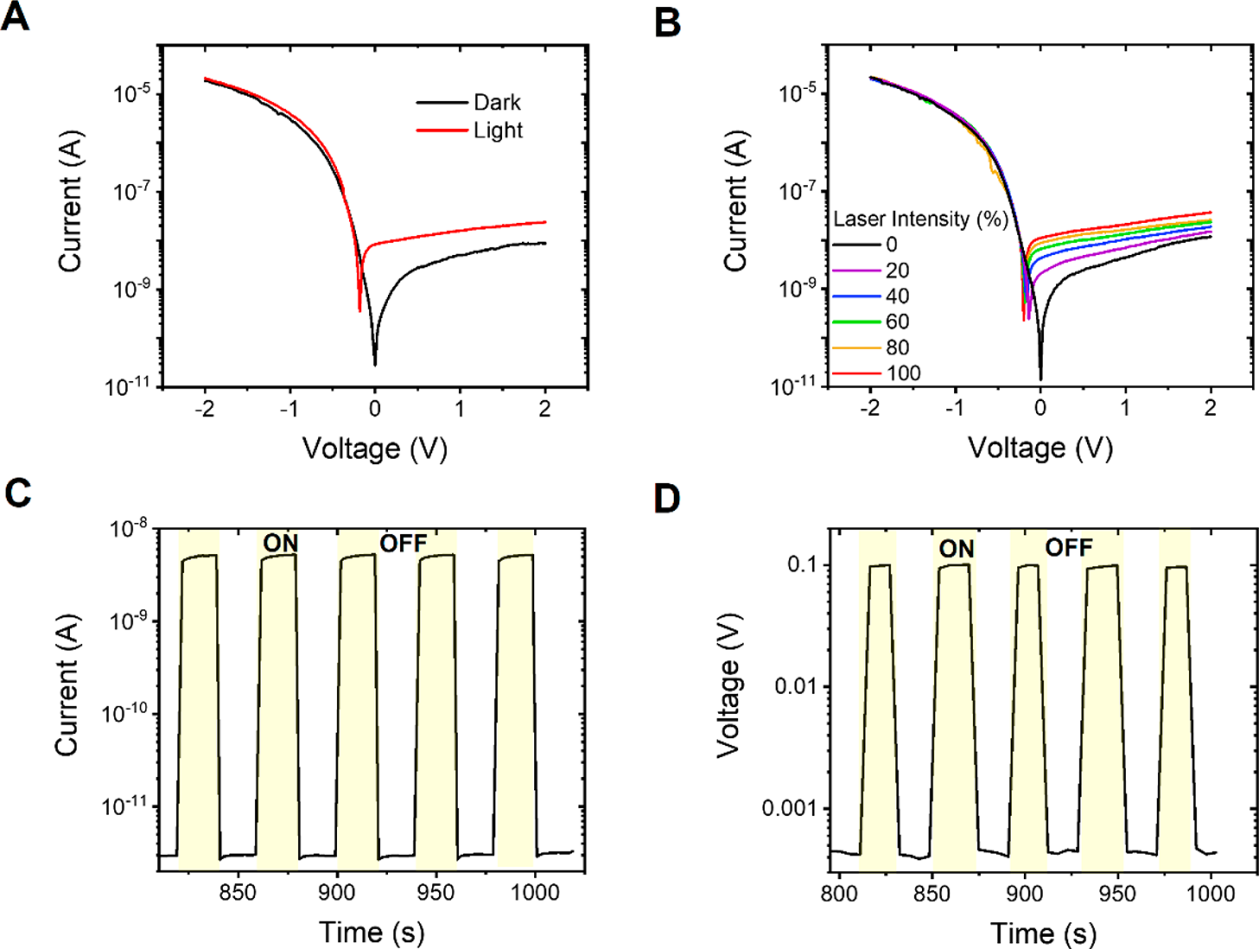
Electrical characterization of the graphene-based diode under dark and light conditions. (A) *I*–*V* characteristics of the diode recorded in dark (black line) and while irradiating the junction with a supercontinuous white laser source (red line). (B) *I*–*V* characteristics of the diode at different laser intensities. (C) Current through the diode monitored while alternating 20 s of dark and 20 s of irradiation (yellow areas) measured at *V* = 0 V. (D) Voltage drop on the diode monitored while alternating 20 s of dark and 20 s of irradiation (yellow areas) measured in self-powered mode (*I* = 0).

Figure 3A shows the *I*–*V* characteristics of the Gr–Si diode in the dark and under illumination by a supercontinuum white laser. The measurement carried out under light exposure shows that the *I*–*V* curve is shifted to the left, compared to the one obtained under dark conditions, indicating an evident photovoltaic effect. When the Gr–Si junction is irradiated by light, the Si substrate absorbs photons with energy higher than the band gap, and electron–hole pairs are generated. These pairs are quickly separated by the built-in field of the Gr–Si junction formed in the depletion zone, and electrons and holes are collected at the electrodes, generating the observed photocurrent. Moreover, electrons from graphene can also be excited over the Schottky barrier and contribute to the photocurrent.^13^ The parameters commonly used to evaluate the performance of a photovoltaic device are the open-circuit voltage (*V*_oc_), the short-circuit current (*I*_sc_), the fill factor (FF), and the power conversion efficiency (PCE). *V*_oc_ corresponds to the maximum voltage available from the photovoltaic device, and it occurs when the current is zero. It matches the amount of direct polarization on the junction due to the illumination, and it has a logarithmic dependence on the intensity of light. In our case, we obtain *V*_oc_ ∼ 0.2 V, comparable to values reported from graphene–silicon heterojunction-based devices.^41,42^*I*_sc_ is the current measured when the voltage across the device is zero. It is entirely due to generation and collection of carriers generated by the light, and it corresponds to the maximum current achievable by the photovoltaic device. We observe a significant current of a few nA, confirming the good photovoltaic performance of our diodes. FF is defined as the ratio between the maximum power of the device (*I*_max_·*V*_max_) and that of the one of the ideal device (FF = (*I*_max_·*V*_max_)/(*I*_sc_·*V*_oc_)), and in our case, it results in FF ∼ 40%. PCE is defined as the ratio between the energy output from the device and the intensity of the incident light (*I*_max_·*V*_max_/*P*_light_). Despite being strongly influenced by several factors, such as the intensity of light and the temperature of the device, the PCE is the most commonly used parameter to compare the performance of different photovoltaic devices. In our case, we obtain a PCE of ∼2%, which is a good achievement compared to values commonly reported in the literature for CVD graphene with no further processing (see section III in the Supporting Information), considering the low intensity of the incident light and that our devices have not been subjected to any engineering process.^42,43^ In particular, our results indicate that Si–Gr devices with good performance can be achieved using printed graphene films; i.e., parameters such as sheet resistance and mobility have little effect on the device performance, as also theoretically shown in ref (44), hence enabling integration of graphene inks, instead of CVD graphene, into Si technology.

Figure 3B shows the *I*–*V* characteristics of the diode obtained tuning the emission power of the laser from 0 to 100 μW/cm^2^. The photoresponse of the device ((*I*_light_ – *I*_dark_)/*I*_dark_) calculated at *V* = 2 V, as shown in in section III in the Supporting Information, follows a linear relationship which ensures that a maximum light effect is reached at the maximum laser power. Therefore, we further investigated the optical response of the devices setting a power of 100 μW/cm^2^ to test the Gr–Si device in photocurrent and photovoltage modes. Figure 3C,D shows the device current/voltage recorded switching on and off the laser every 20 s, at *V* = 0 V and *I* = 0 A, respectively. It is observed that, under illumination, the device generates both a current and a voltage; i.e., it can be operated in a self-powered mode for over 15 min of continuous cycles. Dark–light–dark sequences have been used to estimate the characteristic time for the electron–hole pairs’ photoexcitation and recombination, respectively. In Supporting Information section III, we show that the electron–hole couple generation and recombination time are 0.38 and 0.25 s, respectively.

**Figure 4:**
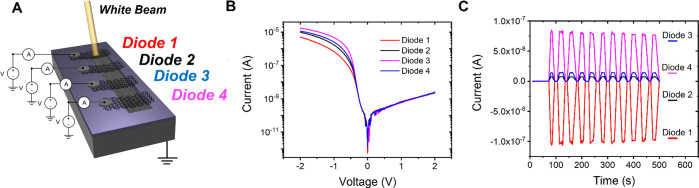
Spatially selective light detector. (A) Schematic of the photodetector and of the electrical setup showing four graphene lines printed on the same silicon substrate. The laser is focussed on diode 1. (B) *I*–*V* characteristics of the diodes 1−4 recorded in the dark and at room temperature. (C) Current through the diodes monitored while alternating 20 s of dark and 20 s of irradiation on diode 1 measured at *V* = 0 V.

The potential of the “scratch and print” approach proposed in this work enables a network of diodes to be made in a very simple and fast way. To demonstrate this idea, we realized a device with four parallel diodes (called diode 1, diode 2, diode 3, and diode 4, Figure 4A) to be used as a spatially selective light detector. Four graphene lines have been printed on a common silicon substrate, which was prescratched before printing and separately connected to the source-meter units (Figure 4A). Figure 4B shows the *I*–*V* characteristics of the four diodes simultaneously measured under dark conditions: all four diodes show the same electrical behavior, with slightly different current values mainly due to different series resistances. To test the device as a photodetector, the current in the four diodes was measured while setting a common bias of *V* = 0 V on all devices and alternating 20 s in the dark and 20 s of light irradiation only on diode 1. Figure 4C shows the current in the four diodes as a function of time for several ON/OFF cycles. When the light is on, the current in diode 1 increases with a negative sign. A current of equal value, but of opposite sign, splits into the other three diodes, and the diode with the lower resistance (diode 3) receives the higher current. This effect can be explained as follows: when the junction between Gr–Si is irradiated, electron–hole pairs are formed; as in the case of the single diode, the built-in potential splits the charges, causing electrons to be attracted to the graphene of diode 1, leading to the observed increase in its current. The remaining excess holes are not attracted to the substrate but to the other three graphene lines, which behave as current sources, giving rise to the opposite sign currents. This is due to the fact that photogeneration occurs mainly at the silicon surface,^45^ so the least resistive path for the holes is the one through the graphene and not the one through the substrate. This conduction mechanism is confirmed when testing the photodetector using only two or three diodes, as shown in section III of the Supporting Information. The observed feature allows the photodetector to be used in self-powered mode. Indeed, it is possible to identify the irradiated area of the chip simply by looking at the current sign. Furthermore, by illuminating a single junction, it is possible to generate current in all of the other diodes, considerably improving the photovoltaic efficiency of the device as a consequence of an improved charge collection. The resolution of our detector is in the range of few millimeters (i.e., the distance between the diodes) but is limited by the size of the laser spot. Indeed, the resolution of the photodetector could easily be further improved by using a focused laser beam.

### Si-Based Integration

**Figure 5:**
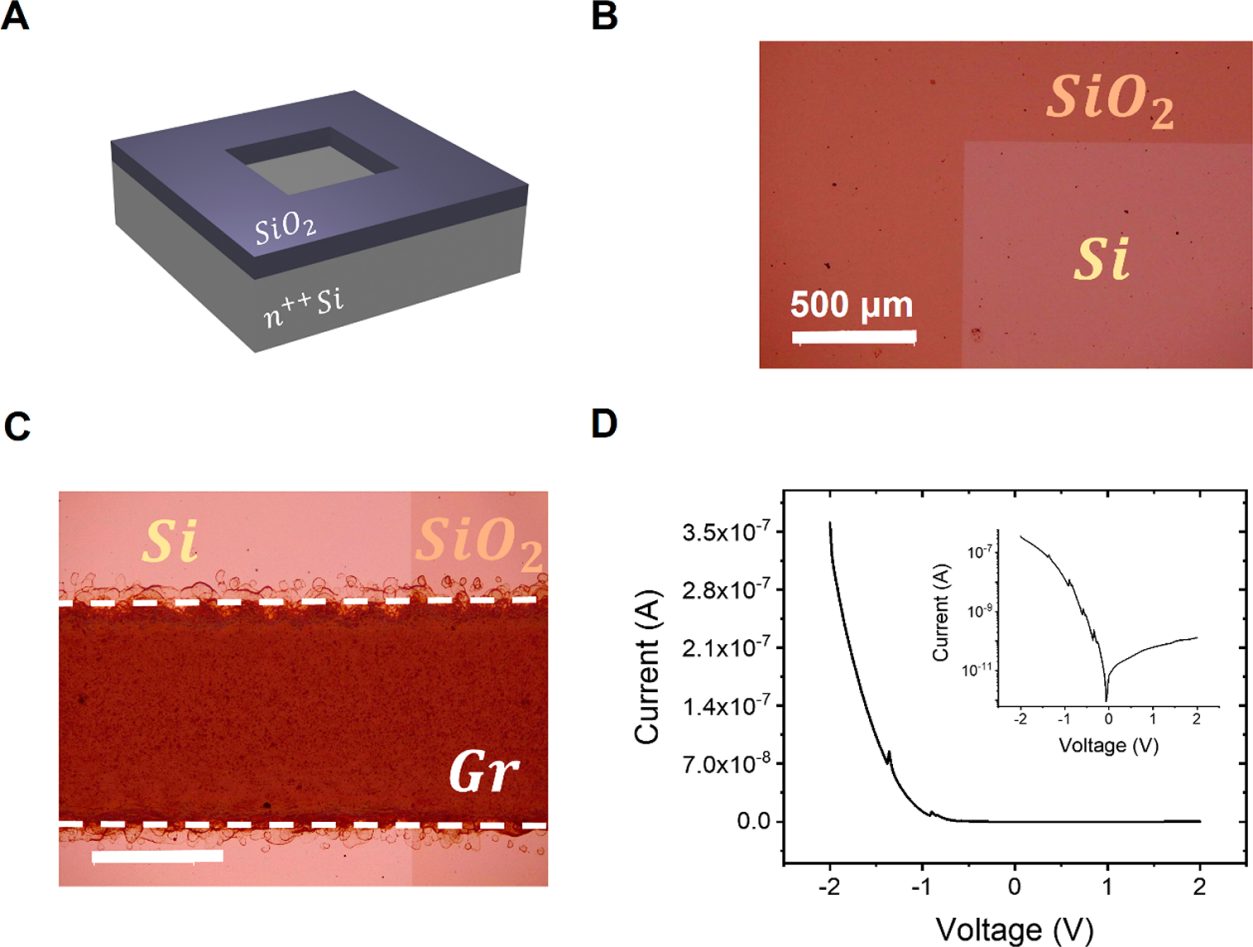
Printed diode on pre-etched silicon wafer. (A) Schematic of the prepatterned silicon substrate showing a chemical etched region in the SiO_2_. (B) Optical image of the substrate showing the edge between the SiO_2_ and the exposed Si. (C) Optical image of the graphene line partially printed on the SiO_2_ and partially on the exposed Si surface. (D) *I*–*V* characteristic of the diode recorded in air and at room temperature.

While the scratching and print is simple and quick, it is likely not compatible with the typical processes used to make silicon chips. It is clear that in a BEOL process, a chip is application-specific. Here, we demonstrate that the diodes can be made onto industrial-type silicon substrates, patterned with standard etching methods used in clean rooms. Figure 5A shows a schematic of the substrate structure, where an area of 9 mm^2^ of thermally grown SiO_2_ was wet etched to expose the Si surface. An optical image of the obtained structure is reported in Figure 5B. The graphene ink is printed on the substrate by setting the same parameters used for the devices made on the scratched silicon. Figure 5C shows an optical image of the junction. The *I*–*V* characteristic of the Gr–Si device recorded in the dark at 300 K, and the atmosphere pressure reveals that the diode made on the patterned substrate has the same electrical behavior of the one realized on the scratched silicon, as shown in Figure 5D; i.e., a typical diode behavior is observed with an ON/OFF ∼ 10^3^ at *V* = ±2 V.

## Conclusions

We demonstrate a simple and quick method, based on inkjet printing of graphene, to realize graphene–silicon Schottky diodes. The obtained devices show a good rectifying behavior of about 3 orders of magnitude in the ±2 V range and a marked photovoltaic effect when exposed to white light. Figures of merit such as an ideality factor of about 2.7 and a Schottky barrier height of about 0.3 eV along with an open-circuit voltage of about 0.2 *V* and a power conversion efficiency of 2% make the devices suitable for both electronic and optoelectronic applications. The possibility of realizing large-area pixeled photodetectors has been exploited. We show that the same diode can be fabricated on a silicon substrate prepatterned ad hoc with standard clean-room-based etching techniques, hence demonstrating that our approach is compatible with BEOL processes for large-scale device production. These results demonstrate that inkjet printing can be a cost-effective and scalable method for the integration of graphene in the modern silicon technology.

## Methods

### Materials

Bulk graphite powder was purchased from Graphexel or Sigma-Aldrich (99.5% grade). Doped n-Si (100) wafers with a resistivity of ∼10 Ω·cm, corresponding to a phosphorus dopant density of ∼4.5 × 10^14^ cm^–3^, covered with 300 nm thermally grown SiO_2_, were purchased from Siegert Wafer. For the patterned substrates, a 3 × 3 mm^2^ trench was obtained by chemical–mechanical polishing and wet etching of the silicon oxide.

### Ink Preparation and Printing

The water-based inks were prepared as described in ref (20). The graphene ink is composed of nanosheets with an average lateral size and thickness of ∼170 and ∼6 nm, respectively.^46^ Note that the nanosheets are exfoliated with noncovalent functionalization in water, so the thickness of the nanosheet also includes a contribution from the stabilizer molecules adsorbed on both sides of the flake.^46^ Previous characterization shows that the nanosheets have high crystallinity, and they are C–O free.^47^ A Varian Cary 5000 UV–vis spectrometer was used to measure the absorption spectrum of the ink. The Lambert–Beer law was used to obtain the concentration of graphene by using an absorption coefficient of 2460 measured at 660 nm.^48^ Immediately before printing, the Si substrates were cleaned with acetone, isopropyl alcohol, and DI water. A Dimatix Materials Printer 2850 (Fujifilm) was used to print 2 mg mL^–1^ graphene ink on top of the scratched area. The spatial resolution of the printer is ∼50 μm on silicon. The platen temperature was set at 42 °C, and cartridges with a droplet volume of 10 pL were used, setting a drop spacing of 35 μm and 80 printing passes.

### Device Characterization

A Bruker Dektak XT system was used to measure the roughness of the substrate and the thickness of the printed graphene (section I in the Supporting Information). Electrical measurements were performed with a Janis probe station (Janis ST-500 probe station) equipped with four nanoprobes connected to a Keithley 4200 SCS (semiconductor characterization system), working as source-measurement unit with current sensitivity better than 1 pA. The electrical measurements were performed in a pressure range from 1 bar (room pressure) to 10^–5^ mbar, obtained by evacuating the air in the chamber by a rough and a turbo pump, and at different temperatures from 220 to 400 K. The photoconductivity was tested by irradiating the devices with a supercontinuous white laser source (NKT Photonics, Super Compact, wavelength ranging from 450 to 2400 nm).

## Supporting Information Available

Substrate and graphene roughness analysis (section I); equivalent circuits of the used models (section II); Cheung’s analysis and parameters evaluation (section II); Richardson’s plot (section II); pristine undoped graphene/n-type silicon band structure (section II); reproducibility tests on different devices (section II); comparison with diodes formed with different metals (section II); photoresponse evaluation (section III); diode array photodetector tests (section III); photovoltaic performances comparison with literature (section III) (PDF)
