# Supplementary material for: Etch and Print: Graphene-Based Diodes for Silicon Technology
Source: ACS Nano. 2022 Dec 7;17(2):1533–40. doi: 10.1021/acsnano.2c10684 (PMC9878974; doi:10.1021/acsnano.2c10684)
Supplement: Supplementary file 1 — nn2c10684_si_001.pdf [file nn2c10684_si_001.pdf]

Supporting Information

Etch and Print: Graphene-based Diodes for Silicon-technology

Alessandro Grillo[1], Zixing Peng[1], Aniello Pelella[2], Antonio Di Bartolomeo[2], Cinzia Casiraghi[1,]*

[1] *Department of Chemistry, University of Manchester, Manchester, M13 9PL, UK*

[2] *Physics Department "E. R. Caianiello", University of Salerno, via Giovanni Paolo II n. 132, Fisciano, 84084, Salerno, Italy*

Corresponding author email: *cinzia.casiraghi@manchester.ac.uk

Content:

I Substrate and graphene roughness analysis

II Equivalent circuits and diode modelling

III Photoresponsivity and diodes array optical response

I Substrate and graphene roughness analysis

Figure S1A shows a map of the graphene-silicon junction morphology obtained through a Bruker Dektak XT system. The measured root mean square (RMS) roughness of printed graphene film is 600 nm onto the pristine SiO_2 and 950 nm for the graphene printed on scratched silicon. Single line profilometry measurements have been also carried out on different points of the graphene printed line. The results are consistent with those obtained through 3D mapping, as shown in the figures S1B and S1C.

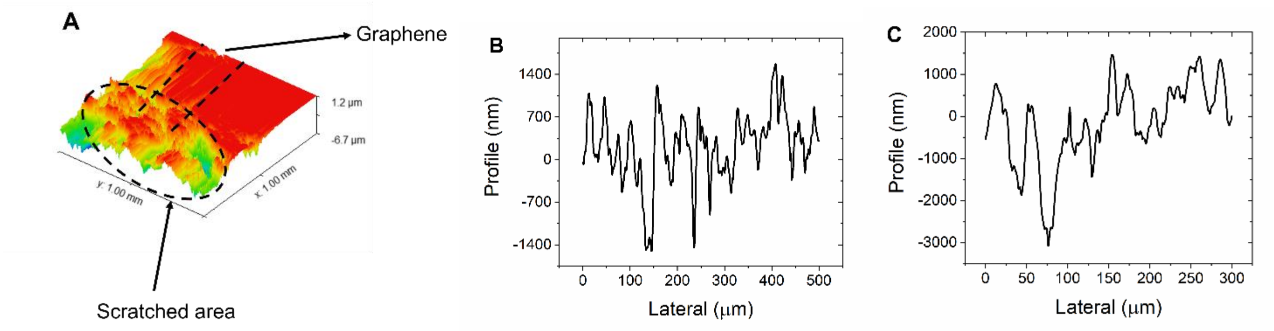

Figure S1. A) 3D plot of the graphene-silicon junction showing both the substrate and the printed graphene. B) Single line profilometry measurement of the graphene onto the pristine SiO_2. C) Single line profilometry measurement on the graphene onto the scratched Si.

II Equivalent circuits and diode modelling

Figures S2A, S2B and S2C show the equivalent circuits of the theoretical models used to study the current in the diode. Figure S2A represents the diagram of an ideal diode in which all the current flows through the graphene-silicon junction, modelled by eq. 1 (main text). Figure S2B shows a more realistic model that takes into account the possible presence of a series resistance R_s, described by eq. 3 (main text). This equivalent circuit is also used to estimate the value of the series resistance, the ideality factor and the Schottky barrier height through the Cheung's method, as

described below. Figures S2C shows the equivalent circuit modelled by eq. 6 (main text), which takes into account possible leakage currents through the oxide and the substrate edges. Figure S2D shows the derivative of the voltage respect to the natural logarithm of the current, $\frac{dV}{dln(I)}$, as function of the current, superimposed to the linear fit used to estimate the series resistance and the ideality factor, through the eq. 4 of the paper. Figure S2E shows the eq. 5 of the paper, i.e. $H(I)$, obtained from the Cheung's method, superimposed to the linear fit used to obtain the Schottky barrier height. Figure S2G shows the band diagram of a standard junction between undoped graphene and n-type silicon. The Schottky barrier height φ_{b0} is give from the difference between the graphene work function ϕ_g and the silicon electron affinity χ_{Si}. Finally, the $I-V$ characteristics measured at different temperatures have been used to obtain the Schottky barrier height and, as byproduct, the effective Richardson constant. First, the linear fittings of the forward current were used to extrapolate the current I_0 at $V = 0\ V$. According to Equation 2, φ_{b0} and A^* can be obtained from the slope and intercept of $ln\ (I_0/T^2)$ versus $1/kT$ (also known as Richardson's plot). We obtained $\varphi_{b0} = 0.04\ eV$ and $A^* = 1447\ A * cm^{-2} * K^{-2}$ as shown in Figure S2F.

To verify the reproducibility of the results, the measurements and analysis were repeated on 12 devices. Figure S3A to S3L show the $I-V$ characteristics of the 12 Gr-Si diodes recorded under dark conditions at $300\ K$ and atmosphere pressure. All the devices show a rectifying behaviour. Table S1 shows the ON/OFF ratio, the Schottky barrier height and ideality factor values obtained for the different diodes. All devices show the same electrical behaviour with slight differences in the figures-of-merit due to the presence of local defects.

As comparison, we used scratched silicon substrates to realize junctions with different metals. Figures S4A, S4B and S4C show the I-V characteristics obtained from the junction with a W-tip, with the silver paste and with a printed silver line. In all three cases we obtained a symmetric characteristic, testifying the absence of a Schottky junction. These results further demonstrate that

the good optoelectronic performances of the diodes reported in the paper are due to the interaction between the printed graphene and the silicon.

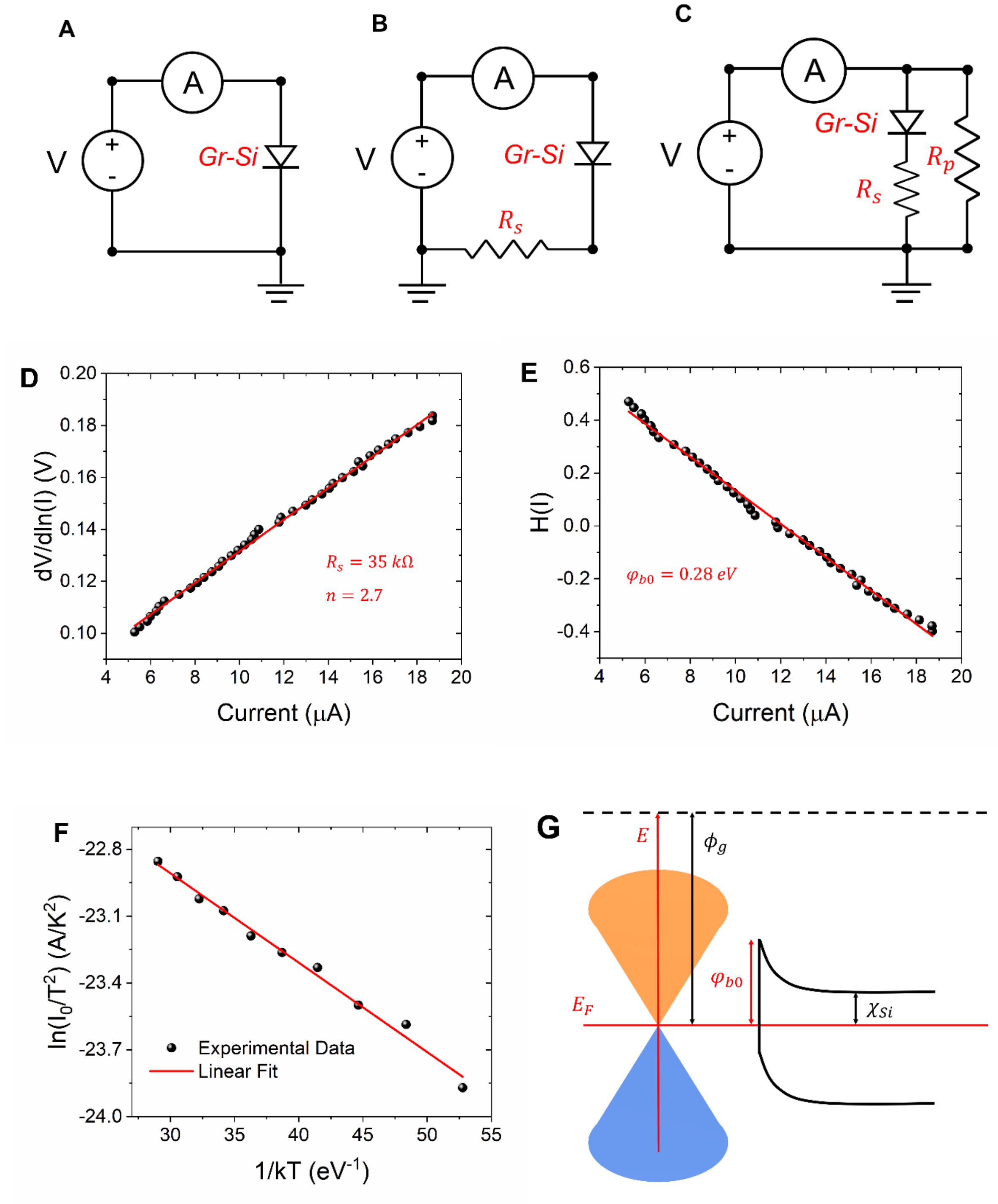

Figure S2. A) Ideal diode model considering that all the current flows through the graphene-silicon junction. B) Model with a diode and a series resistance used to evaluate the ideality factor and the Schottky barrier height. C) Model with both a series and a parallel resistance considering possible

leakage currents through the oxide and the substrate edges. D) Derivative of the voltage respect to the natural logarithm of the current used to estimate the series resistance and the ideality factor. E) $\mathrm{H(I)}$ from the Cheung's method used to evaluate the Schottky barrier height. F) Richardson's plot used to evaluate the Schottky barrier height and the effective Richardson's constant. G) Band alignment for an ideal junction between undoped graphene and n-type silicon.

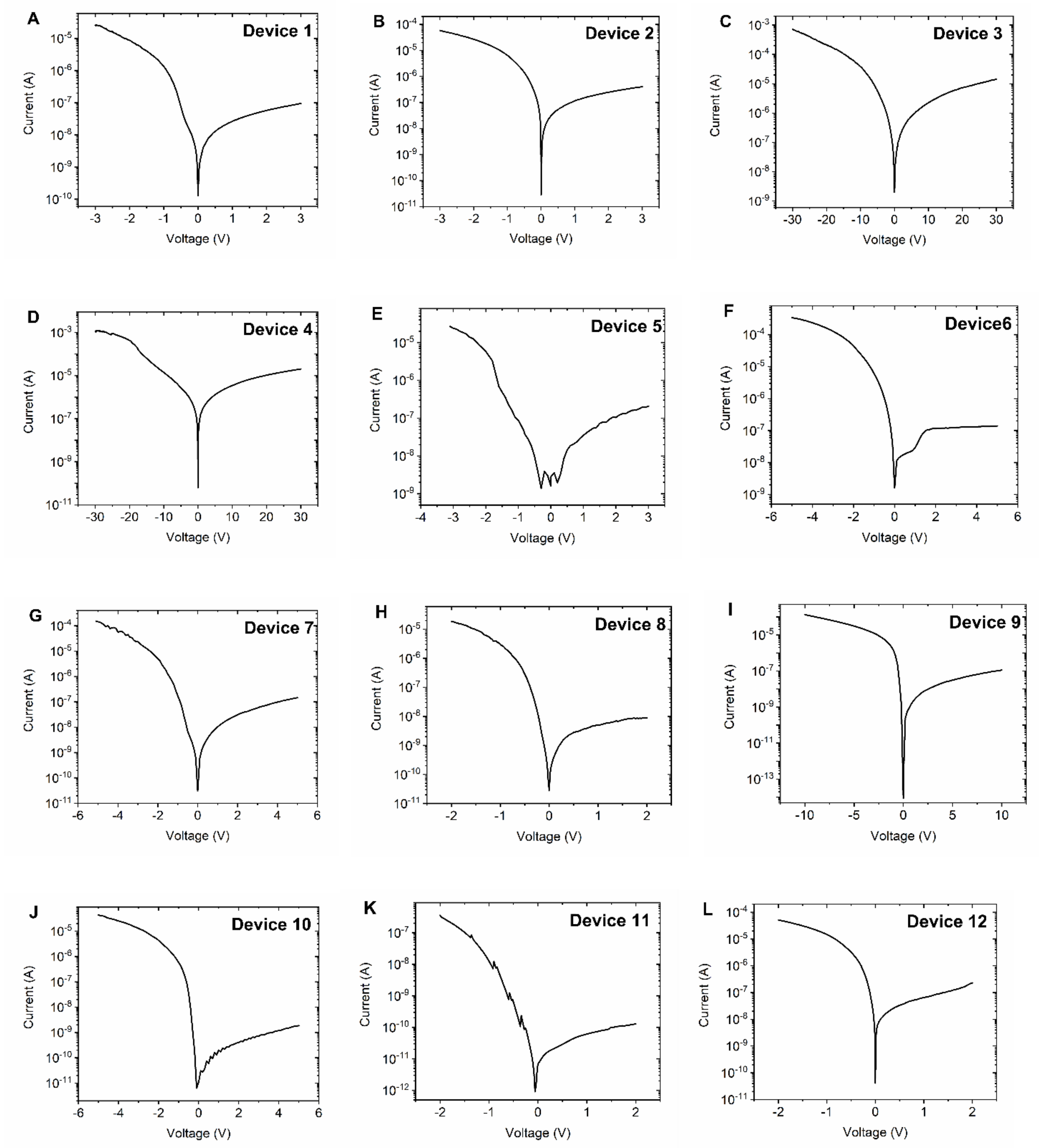

Figure S3. A) to L) $I - V$ characteristic of the 12 Gr-Si junctions investigated in this work recorded under dark conditions at $300\ K$ and atmosphere pressure.

Device Number	1	2	3	4	5	6
ON/OFF	276	143	51	56	165	2409
$\varphi_{B0}(eV)$	0.28	0.53	0.60	0.33	0.16	0.23
n	5.3	3.7	22	30	6.9	4.6

Device Number	7	8	9	10	11	12
ON/OFF	1012	2127	8098	23378	2841	225
$\varphi_{B0}(eV)$	0.38	0.79	0.69	0.28	0.31	0.22
n	2.1	2.9	8.4	2.1	3.5	4.6

Table S1. The table reports the ON/OFF ratio, the Schottky barrier height and the ideality factor measured for all the 12 different graphene-silicon junctions under test.

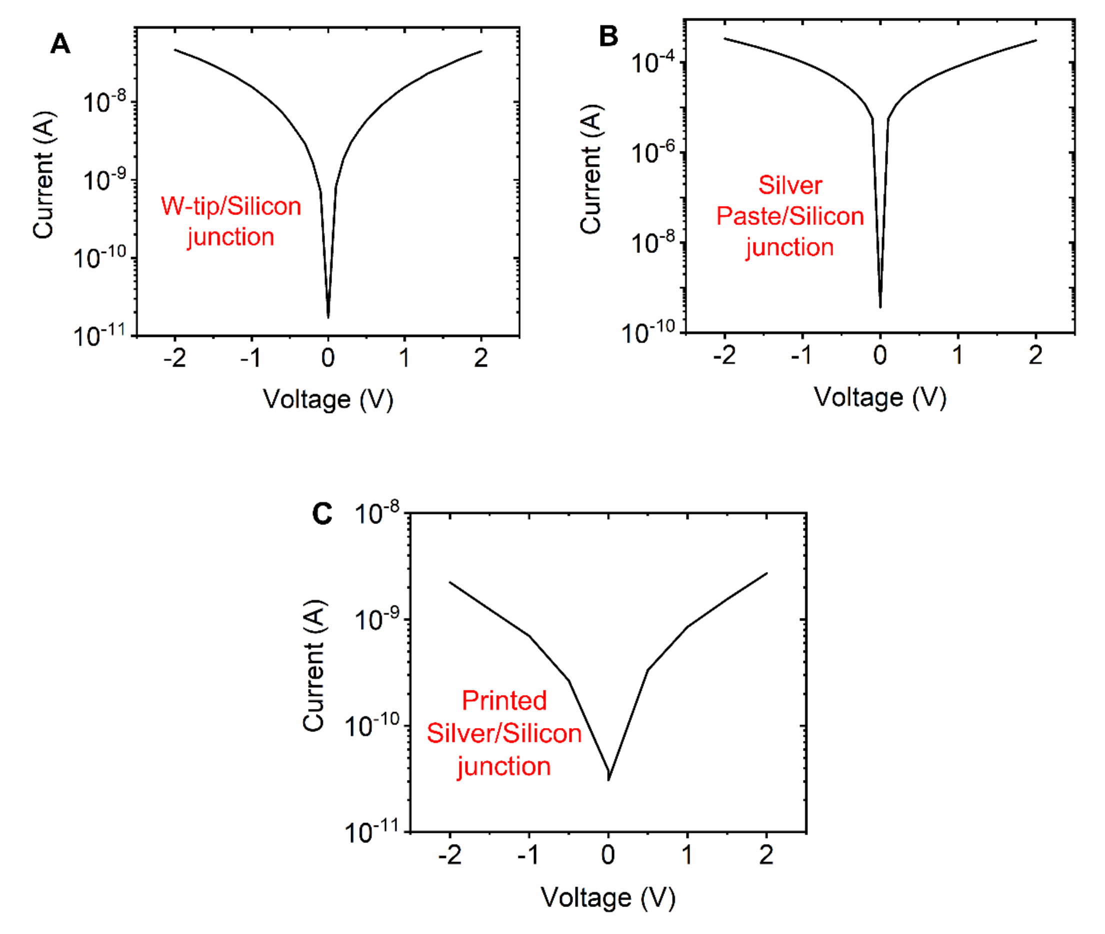

Figure S4. A),B) and C) $I - V$ characteristic of the junction between Silicon and W-tip, silver paste and printed silver, respectively.

III Photoresponsivity and diodes array optical response

Figure S5A shows the photoresponse of the device ($(I_{light} - I_{dark})/I_{dark}$) calculated at $V = 2\ V$. It reveals a linear dependence on the intensity of the incident light, so the following optical measurements were carried out by setting a maximum power of 100% corresponding to $100\ \mu W/cm^2$.

Figures S5B and S5C show the photodetector tests carried out on the diodes array structure reported in Figure 4a of the paper. The current has been monitored while setting a common bias

$V = 0\ V$ to two and three diodes, respectively, and alternating 20 seconds in dark and 20 seconds of light irradiation only on diode 1. The observed results are consistent with those reported for four diodes, shown in the paper. Indeed, in the case of two diodes (Figure S5B) the current is equally divided in the two junctions, while in the case of three diodes (Figure S5C), the sum of the currents generated in the two diodes kept in dark is equal to that generated in the diode irradiated by light. The raising and decay currents associated with the electron-hole pairs photo-generation and recombination follow an exponential law: $I = I_O + A * exp\left(-\frac{t}{\tau}\right)$ where τ represents the generation/recombination constant. By taking the natural logarithm to both left and right terms, the characteristic time constants can be obtained from the slope of linear fits, associated to the increase in current after the dark-light switch and to the decay in current, after the light-dark switch. Figure S5D plots ln(I) vs t for a single dark-light-dark cycle. From the linear fits we obtained, $\tau_1 = 0.38\ s$ for the electron-hole generation and $\tau_2 = 0.25\ s$ for the electron-hole recombination.

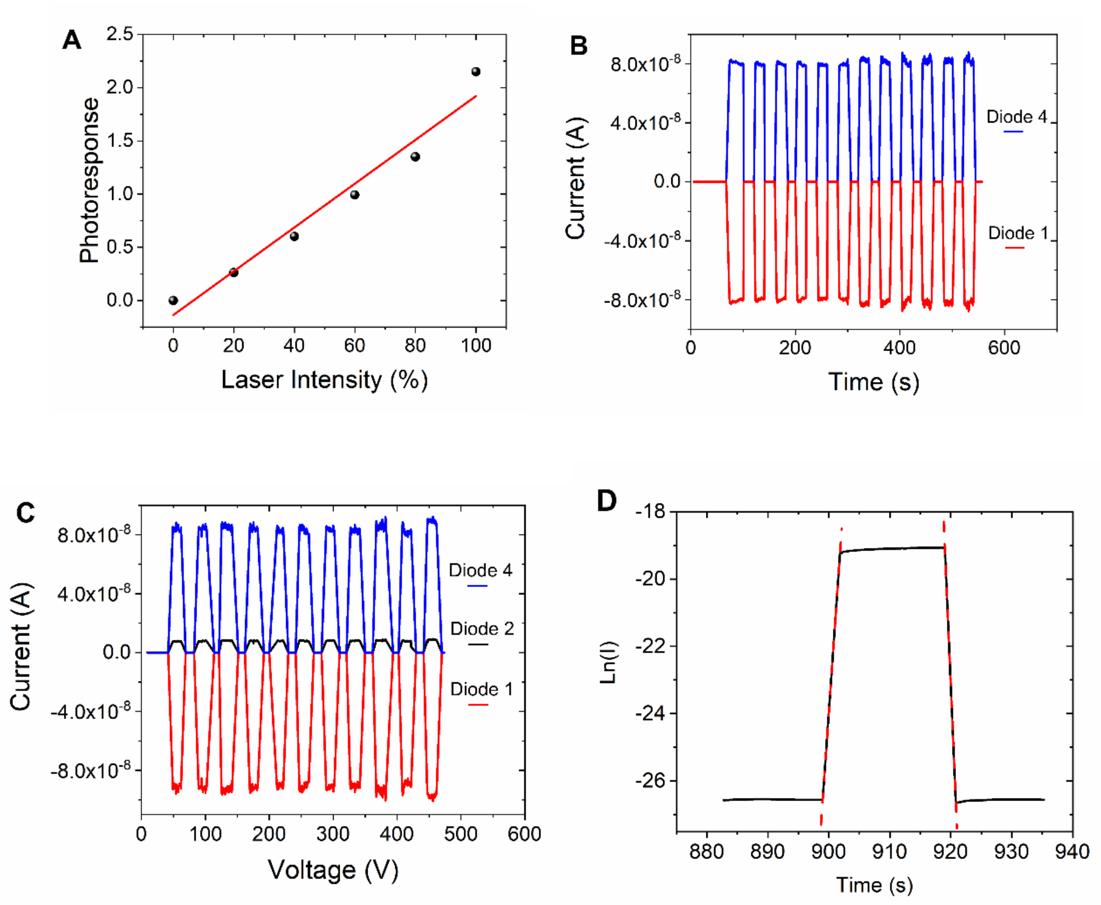

Figure S5. A) Photoresponse of the graphene-silicon junction as function of the laser intensity. B) and C) Current through the diodes monitored while alternating 20 s of dark and 20 s of irradiation on diode 1 measured at $\mathrm{V} = 0\ \mathrm{V}$ using two and three diodes, respectively. D) Single dark-light-dark cycle used to evaluate the speed response of the diode obtained from the linear fit of the natural logarithm of the current as function of the time (superimposed red dashed lines).

Finally, to evaluate the photovoltaic performance of our diode we compared the power conversion efficiency (PCE), the open-circuit voltage ($\mathrm{V_{OC}}$), the short-circuit current ($\mathrm{I_{SC}}$) and the filler factor (FF) achieved in this work with that reported in the literature for similar devices, as showed in Table S2. The obtained PCE is comparable to that achieved from not optimized devices, and further improvements are expected if well assessed techniques such as molecular doping or antireflection coating are applied, confirming the good optical response of our diodes.

**Graphene type; Processing**	**Structure**	$\boldsymbol{PCE}$ (%)	$\boldsymbol{V_{OC}}$ ($\boldsymbol{V}$)	$\boldsymbol{I_{SC}}$ ($\boldsymbol{mA/cm^2}$)	**FF (%)**	**Ref.**
Inkjet Printing; No processing	$Gr - Si$	2	0.2	70000	40	This work
CVD graphene; No processing	$Gr - Si$	1.65	0.42	6.5	56	1
CVD graphene; Chemical doping through HNO_3	$HNO_3 - Gr - Si$	9.27	0.55	17.18	66	2
CVD graphene; Molecular doping through $SoCl_2$	$SOCl_2 - Gr - Si$	5.95	0.55	17.92	60.6	3

CVD graphene; Colloidal antireflection coating	$HNO_3 - TiO_2 - Gr - Si$	14.5	0.61	32.7	72	4
CVD graphene; Optimized oxide thickness, chemical doping and antireflective coating	$Gr - SiO_2 - Si$	15.6	0.51	28	-	5

Table S2. The table reports the power conversion efficiency, the open-circuit voltage, the short-circuit current and the filler factor obtained in this work compared to the ones from similar devices in literature.

References

(1) Li, X.; Zhu, H.; Wang, K.; Cao, A.; Wei, J.; Li, C.; Jia, Y.; Li, Z.; Li, X.; Wu, D. Graphene-On-Silicon Schottky Junction Solar Cells. *Adv. Mater.* **2010**, *22* (25), 2743–2748. https://doi.org/10.1002/adma.200904383.

(2) Li, X.; Xie, D.; Park, H.; Zhu, M.; Zeng, T. H.; Wang, K.; Wei, J.; Wu, D.; Kong, J.; Zhu, H. Ion Doping of Graphene for High-Efficiency Heterojunction Solar Cells. *Nanoscale* **2013**, *5* (5), 1945. https://doi.org/10.1039/c2nr33795a.

(3) Cui, T.; Lv, R.; Huang, Z.-H.; Chen, S.; Zhang, Z.; Gan, X.; Jia, Y.; Li, X.; Wang, K.; Wu, D.; Kang, F. Enhanced Efficiency of Graphene/Silicon Heterojunction Solar Cells by Molecular Doping. *J. Mater. Chem. A* **2013**, *1* (18), 5736. https://doi.org/10.1039/c3ta01634j.

(4) Shi, E.; Li, H.; Yang, L.; Zhang, L.; Li, Z.; Li, P.; Shang, Y.; Wu, S.; Li, X.; Wei, J.; Wang, K.; Zhu, H.; Wu, D.; Fang, Y.; Cao, A. Colloidal Antireflection Coating Improves Graphene–Silicon Solar Cells. *Nano Lett.* **2013**, *13* (4), 1776–1781. https://doi.org/10.1021/nl400353f.

(5) Song, Y.; Li, X.; Mackin, C.; Zhang, X.; Fang, W.; Palacios, T.; Zhu, H.; Kong, J. Role of Interfacial Oxide in High-Efficiency Graphene–Silicon Schottky Barrier Solar Cells. *Nano Lett.* **2015**, *15* (3), 2104–2110. https://doi.org/10.1021/nl505011f.
